# Effects of subgingival air-polishing with trehalose powder on oral biofilm during periodontal maintenance therapy: a randomized-controlled pilot study

**DOI:** 10.1186/s12903-020-01111-9

**Published:** 2020-04-22

**Authors:** Anne B. Kruse, Rabie Maamar, Dodji L. Akakpo, Johan P. Woelber, Annette Wittmer, Kirstin Vach, Petra Ratka-Krüger, Ali Al-Ahmad

**Affiliations:** 1 Department of Operative Dentistry and Periodontology, Faculty of Medicine, University of Freiburg, Hugstetter Str. 55, 79106 Freiburg, Germany; 2 Private Dental Practice Tasler/Steude, Berlin, Germany; 3 Institute of Medical Microbiology and Hospital Hygiene, Faculty of Medicine, University of Freiburg, Freiburg, Germany; 4 Department of Medical Biometry and Medical Informatics, Faculty of Medicine, University of Freiburg, Freiburg, Germany

**Keywords:** Subgingival debridement, Air-polishing, Trehalose, Microbiological changes, Sonic debridement, Periodontal maintenance

## Abstract

**Background:**

This pilot study was part of a larger study which compared the effect of subgingival air-polishing using trehalose powder with sonic scaling on clinical parameters during supportive periodontal therapy. Within this microbiological part of the investigation subgingival samples were taken from 10 participants to analyze the survival of different bacterial species after the two different treatments as a proof of principle.

**Methods:**

In 10 participants two non-adjacent, single-root teeth requiring treatment (PD =5 mm with bleeding on probing (BOP) or > 5 mm) were selected following a split-mouth design and were treated either with a sonic scaler or air-polishing device and trehalose powder. For persistent pockets (PD =4 mm and BOP or *>* 4 mm), treatment was repeated after 3 months. Subgingival biofilm samples were taken at baseline (BL), subsequently and three and six months after treatment. After determination of the bacterial counts (TBL), isolated bacteria were identified by MALDI-TOF-MS. If unsuccessful, PCR and 16S rDNA sequencing were performed.

**Results:**

In both treatment groups, TBL decreased immediately after treatment remaining at a lower level. This confirms the findings of the larger study regarding clinical parameters showing a comparable effect on PD, BOP and CAL. Immediately after treatment, the diversity of detected species decreased significantly more than in the sonic group (*p* = 0.03). After 3 months, the proportion of Gram-positive anaerobic rods was lower in the air-polishing group (powder/ sonic 7%/ 25.9%, *p* = 0.025). Also, there was a greater reduction of Gram-negative aerobic rods for this group at this time (air-polishing/ sonic − 0.91 / -0.23 Log10 cfu/ ml, *p* = 0.020).

**Conclusion:**

Within the limitations of this study air-polishing and sonic treatment seem to have a comparable effect on the subgingival oral biofilm during supportive periodontal treatment.

**Trial registration:**

The study was registered in an international trial register (German Clinical Trial Register number DRKS 00006296) on 10th of June 2015. HTML&TRIAL_ID = DRKS00006296.

## Background

Long-term success of periodontal treatment is crucially dependent on maintenance therapy with repeated subgingival instrumentation of deepened periodontal pockets [1]. Simultaneously, regular supportive periodontal therapy may reduce tooth loss over the years [2]. Since deeper periodontal pockets might not fully be eradicated during active periodontal therapy, repeated subgingival biofilm removal in those sites is essential during supportive periodontal treatment to disturb dysbiotic biofilms and to overcome inflammation [3, 4]. In this context the use of hand instruments as well as sonic scalers or air-polishing devices could be proven effective for subgingival instrumentation [5–7]. Especially air-polishing has for several years become a promising alternative technique with equivalent clinical results and simultaneously proven lower pain sensation to the patient [5, 8, 9]. Air-polishing leads to an effective but gentle abrasion on the root surface by accelerating powder particles using compressed air. Here, powder properties play an important role to make abrasion more effective and simultaneously be easy on the hard and soft tissue in the periodontal pocket. So far two types of powders are suitable for subgingival air-polishing: glycine and erythritole [5, 6]. Trehalose is a new powder which has not been used in the context of air-polishing in a clinical trial before. It is a disaccharide, which consists of two α, α’- 1,1-glycosidically linked glucose molecules and is classified as a food additive in the USA as well as in Europe [10]. It shows particle diameters of 25–35 μm, has a pH of 6.4 and is highly water soluble (689 g/L) according to the manufacturer. There are indications that the use of air-polishing might be favorable concerning the removal of subgingival microbiota. Concerning reduction of bacterial counts immediately after subgingival instrumentation, a superiority of air-polishing over hand instrumentation could be proven in a clinical trial [7]. However, another investigation after twelve months could find no differences of the total bacterial count between air-polishing and sonic scaling compared to baseline [5]. In terms of suppression of selective periodontal pathogens, a clinical trial could show that 90 days after using air-polishing in the oral cavity including subgingival sites, oral mucous membranes and tongue mucosa, the total count of *Porphyromonas gingivalis (P. gingivalis)* was significantly lower than for the group that received only hand instrumentation at subgingival sites. For *Tannerella forsythia (T. forsythia)* however, bacterial counts were favorable for air-polishing after ten days returning to baseline values after 90 days [11]. Also, in patients without periodontitis, an adjunctive supragingival full-mouth-disinfection by air-polishing could lower counts of red complex bacteria like *P. gingivalis* and *T. forsythia* after nine days, but returning to baseline values after six and twelve weeks [12]. In a consensus conference in 2017 it was summarized, that subgingival air-polishing in periodontal pocket depths up to 9 mm is more effective in the removal of biofilm than hand instrumentation or (ultra-) sonic scalers [13].

The detection of subgingival microbiota has undergone a tremendous development over the years. Not least by decoding the oral microbiome, new dimensions of detection and investigation of disease specific clusters have emerged.

Besides the microbiological culture technique, FISH, PCR and Checkerboard DNA-DNA Hybridization are established research methods [14–16]. Recently, next-generation high throughput sequencing has led to a revolution in the evaluation of oral biofilm. This method is characterized by high automatability and allows sequencing a large number of DNA samples in parallel [17]. However, the above-mentioned methods have the disadvantage, compared to the classical bacterial culture, of not distinguishing between living and dead bacteria. The culture technique is still the reference method to evaluate the survival of microorganisms when new chemical or mechanical eradication methods of the oral biofilm are being tested [18, 19]. Up to now, only other powders such as glycine and erythritol were used for microbiological evaluation of the efficiency of air polishing in periodontitis treatment. Moreover, the microbiological evaluation reported in literature considered only total bacterial counts or the differentiation of red complex members like *Porphyromonas gingivalis* and *Tannerella forsythia*. No comprehensive microbiological evaluation has been conducted by using the culture technique and identification by MALDI-TOF and 16S rRNA sequencing of unidentified isolates, so far.

Therefore, the aim of this study was to determine whether there is a difference in reduction of viable selected periodontal pathogens after subgingival treatment with air-polishing using trehalose powder compared to sonic scaling during supportive periodontal therapy over a time period of 3 and 6 months (primary endpoint). Secondary endpoints were the differences between groups concerning the relation of aerobic and anaerobic species, the distribution of other subgroups of species and the total number of different species at different time points.

## Methods

This clinical trial respected the principles of the Declaration of Helsinki on human experimentation and was carried out in accordance with Good Clinical Practice. The Ethical Committee of the University Medical Center Freiburg verified this trial with a positive vote (EK No. 317/14). All enrolled participants signed an informed consent form as well as a data privacy statement. This report follows the criteria of the CONSORT statement [20].

### Study design

This trial was part of a larger study which focused on clinical parameters (PD, CAL and BOP) comparing two treatment methods, air-polishing using a new trehalose powder and sonic scaling as control. Furthermore, participants rated both treatments in terms of comfort on a visual analogue scale. It was conducted as a single-blinded randomized clinical trial over six months at the Center for Dental Medicine, Department of Operative Dentistry and Periodontology, University Medical Center Freiburg, Germany [9]. Using a split-mouth design, one tooth was treated subgingivally using an air-polishing device with trehalose powder (test group) while another tooth in the opposite quadrant was treated with a sonic scaler (control group). This microbiological part was added as a pilot study, so a subgroup was formed within the clinical study. Microbiological samples from ten patients were analyzed. The samples were taken from the periodontal site with the deepest pocket depth (PD). Samples were taken before and immediately after treatment, and after three and six months. The total number of bacteria and the change in the bacterial composition were analyzed. All patients received professional supragingival tooth cleaning after three months. Additionally, a follow-up treatment of persisting pockets with PD *>* 4 mm or 4 mm and bleeding on probing took place after three months. Treatment was performed by the same method used at baseline.

### Participants

Fifty-two individuals were recruited during periodontal maintenance therapy from the patient population at the Department of Dental Medicine, University Medical Center Freiburg (see Fig. S1 CONSORT Flow Diagram). All participants needed to meet the following inclusion criteria:

- chronic periodontitis patients undergoing maintenance therapy [21].
- completed cause-related phase of periodontal therapy within the last 2 years.
- 2 single-rooted, non-adjacent teeth from different quadrants with PD = 5 mm and positive bleeding on probing or > 5 mm with or without positive bleeding on probing [22–24].
- no systemic antibiotic treatment during the last 12 weeks.
- good general health (no major disease, e.g., untreated heart disease, poorly controlled diabetes, HIV, cancer, hemorrhagic conditions).
- no pregnant or breastfeeding women.
- smokers were included to a maximum of 30%.

Out of all included participants, ten were randomly chosen for microbiological examination including a maximum of 20% smokers.

### Intervention

In the test group a new trehalose powder (Lunos® Prophylaxis Powder Perio Combi, Dürr Dental SE, Bietigheim-Bissingen, Germany) was applied using an air-polishing device.

(Perio-Flow® handpiece with Perio-Flow® Nozzle EMS, Nyon, Switzerland). For the control group a sonic device was used for subgingival instrumentation (Sonic Flex, KaVo, Biberach/Riß, Germany).

The procedure assigned by randomization was carried out on the respective teeth for 20 s each, irrespective of air-polishing or sonic instrumentation. 3 months after intervention all participants received oral hygiene instructions by the same dentist and received professional supragingival cleaning. If there was a need for follow-up treatment (PD = 4 mm and positive BOP or PD > 4 mm), the assigned intervention was repeated in terms of a state of the art supportive periodontal treatment [1, 24].

### Microbiological examination

#### Collection and storage of samples

Following supragingival professional tooth cleaning and recording of clinical parameters, the microbiological samples were taken from the reference periodontal pocket using 40/04 sterile paper tips. The withdrawals were performed at baseline (before and immediately after treatment), at three, and six months. The samples were preserved in 0.75 mL reduced transport fluid and stored in a freezer at − 80 °C [25].

#### Processing of the samples

In a first step, the frozen samples were thawed in a water bath at 36 °C and then homogenized for 45 s by vortex. Subsequently, a dilution series was carried out and the samples were applied on three different nutrient media in order to culture the individual colonies of the different bacterial species. The following agar plates and liquid media were used:

- Columbia Blood Agar (CoBl): for the cultivation of aerobic and facultative anaerobic bacteria
- Yeast cysteine ​​blood agar (HCB): for culturing anaerobic bacteria
- Trypticase Soy Bacitracin Vancomycin Selective Agar (TSBV): for the cultivation of
  *Aggregatibacter actinomycetemcomitans*
- Dilution medium (PY): this medium was used to prepare the dilution series

#### Isolation and identification of bacterial species

After an incubation period of 4–5 days for CoBl plates and TSBV Selective agar plates at 36 °C in a CO_2_ cabinet or an incubation period of 10–12 days for HCB plates at 36 °C under anaerobic conditions, the colony forming units (CFU) on agar plates were counted. Single colonies were assessed for shape and color. This served the temporary classification of the bacterial species. The number of bacteria was determined as follows:

#### Bacteria number / mL = colony number x dilution grade

For the final identification of the bacteria, the individual colonies were subcultured to obtain pure cultures of the germs.

The final identification of the bacteria was mainly done using MALDI-TOF MS (Matrix Assisted Desorption Ionization Time of Flight Mass Spectrometry) analysis in a MALDI Biotyper Microflex LT as described in detail earlier [26]. In brief, bacteria from single colonies were used for MALDI-TOF. The acquisition of the mass spectra was conducted according to the manufacturer’s recommendations. The obtained spectra were compared using the BioTyper 3.0 software with a reference database containing 3740 reference spectra (representing 319 genera and 1946 species). The resulting similarity value was expressed as a log score. Species level identification was indicated by a score of ≥2000, whereas a score of ≥1700 indicated identification on the genus level. No significant similarity of the obtained spectrum with any database entry was obtained by score value under 1700. The procedure was repeated, if the results were questionable.

If identification by MALDI-TOF-MS was unsuccessful, identification by polymerase chain reaction (PCR) and subsequencing of the 16S rDNA genes was used. The identification of bacterial species by 16S rDNA sequencing was conducted as described earlier in detail (Schirrmeister et al., 2009). In brief, DNA was extracted from pure bacterial isolates of Gram-negative bacteria by using lysis buffer (10 mmol/L Tris-HCl buffer, 1 mmol/L ethylenediaminetetraacetic acid, 1% Triton X-100, pH 8.0) and boiling for 10 min. After centrifugation at 12,000 g for 10 min, 1 μL of the supernatant was used for amplification of the 16S rRNA gene. DNA from Gram-positive bacterial isolates was extracted by using the QIAamp DNA Mini Kit (Qiagen, Hilden, Germany) according to the manufacturer’s instructions. 16S rRNA gene amplification was performed in a total volume of 50 μL, containing 5 μL 10X PCR-buffer (Qiagen), MgCl2 (2.5 mmol/L), 200 mol/L of each deoxyribonucleoside triphosphate (dNTP), 2 U Taq Polymerase (Qiagen), and 300 nmol/L of reverse- and forward-primer. For amplification of the 16S rRNA gene, a set of primers (forward-primer TP16U1: 5′-AGAGTTTGATC [C/A]TGGCTCAG-3′ and reverse-primer RT16U6: 5′-ATTGTAGCACGTGTGT [A/C]GCCC-3′) was used. The 1018 base pair-long PCR products were extracted and purified by using the GFX PCR DNA and gel band purification kit (Amersham Biosciences Europe GmbH, Freiburg, Germany). Purified PCR Products were sequenced by using the BigDye terminator kit v1.1 cycle sequencing kit (Applied Biosystem, Darmstadt, Germany) and the ABI 310 Genetic Analyzer (GMI, Inc., Ramsey, MN). TP16U1 was used as a sequencing primer. To identify then bacterial species sequences were analyzed by using the BLAST program from the NCBI (http://www.ncbi.nih.gov/BLAST). This was done in the present work for the identification of only 4 bacterial isolates, which were not identifiable in MALDI-TOF-MS. These bacterial species were *Prevotella tannerae, Anaeroglobus geminatus, Actinomyces* sp*.* oral taxon*, Filifactor alocis.*

#### Blinding and randomization

Participants were randomized to determine which tooth belonged to one of the two treatment arms. A randomization list was computer-generated by a statistician (KV) after participants were enrolled by two dentists (DA and RM). Concealed envelopes for each participant were prepared according to the randomization list. All baseline and follow-up examinations and microbiological sampling were done by the same dentist (DA). Interventions were exclusively undertaken by RM opening the envelope shortly before the treatment to know which method to use in each quadrant. By this procedure DA was blinded as an examiner.

#### Statistical analysis

For descriptive analysis, mean and standard deviation were calculated. Boxplots were used for the graphical presentation. Paired t-tests compared the temporal changes to baseline in log10 transformed bacterial concentration between the two methods and within the two groups. The calculations were done with STATA 15.

## Results

For demographic data see Table 1.

**Table 1:** Demographic data. Values are given as means with standard deviations in parentheses

Participants (*n* = 10)	
Age	61.4 years (±10.6)
Gender	80% male, 20% female
Ethnic group	100% Caucasian
Smoking status	20% smokers mean count of 20.6 packyears (± 18.9)

### Summary of clinical parameters

In order to present the overall picture of the study, the results of the clinical parameters are briefly summarized. There were found no significant differences between test and control group regarding BOP, PD and CAL over 3 and 6 months (see also Table 2). 8 out of 10 participants needed retreatment for both teeth that were included in the study. Regarding discomfort air-polishing showed a significantly lower incidence of pain on a visual analogue scale [1–10] compared to sonic scaling (test 2.33 ± 2.14, control 4.91 ± 2.65, *p* < 0.001). For more detailed information see original publication of Kruse et al. 2019 [9].

**Table 2:** Main clinical results regarding periodontal parameters from original publication of Kruse et al. 2019 [9].

	group	Baseline = BL (*n* = 52)	3 months (*n* = 48)	6 months (*n* = 44)	*p* Value BL vs. 3 months	*p* Value BL vs. 6 months	*p* Value change test group vs. control group (3 months)	*p* Value change test group vs. control group (6 months)
BOP %	test	86.36	59.09	40.91	< 0.001	< 0.001	0.561	0.693
	control	88.64	63.64	34.09				
PD (mm)	test	5.52 (0.93)	4.25 (1.12)	3.66 (0.81)	< 0.001	< 0.001	0.408	0.907
	control	5.55 (0.90)	4.11 (1.08)	3,68 (0.86)				
CAL (mm)	test	6.93 (1.50)	5.80 (1.65)	5.30 (1.52)	< 0.001	< 0.001	0.82	0.062
	control	7.27 (1.80)	6.00 (1.73)	5.84 (1.71)				

*BOP* bleeding on probing, *PD* pocket depth, *CAL* Clinical attachment level

### Total bacterial counts (see Fig. 1)

**Fig. 1:**
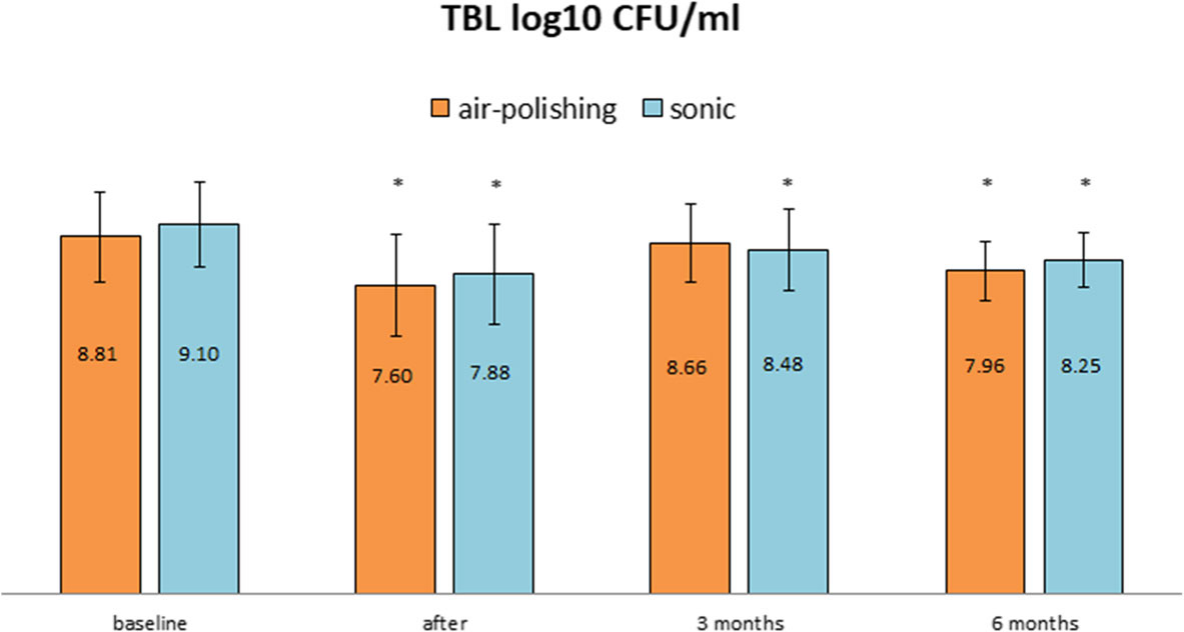
Bacterial counts are given as means in Log10 CFU/mL with standard deviations at different time points (baseline, immediately after treatment, after 3 and after 6 months). There were no statistical differences between the groups. Significant intragroup differences compared to baseline are given for different time points where applicable (* *p* < 0.05)

### All bacteria

The total bacterial counts after treatment significantly decreased by more than one log10 level in both the air-polishing group (*p* < 0.01) and the sonic group (p < 0.01) compared to baseline. Three months after treatment, total bacterial counts increased, but remained below baseline and dropped again at 6 months. Comparing data at baseline and after 6 months, statistically significant differences were found for both groups (air-polishing p < 0.01, sonic *p* = 0.03). However, there was no statistically significant difference between the two procedures at any time point (*p* > 0.05, see Fig. 1).

### Aerobes and anaerobes

**Fig. 2:**
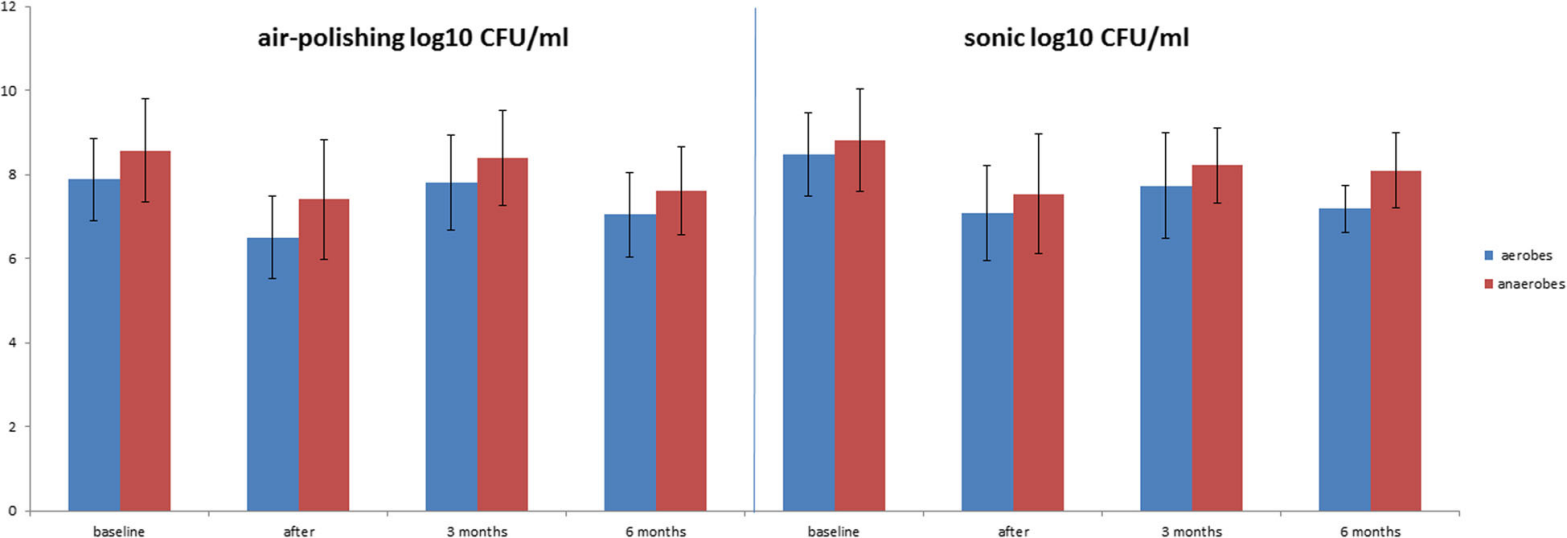
Counts of aerobic and anaerobic species. Values are given as means in Log CFU/mL at different time points (baseline, immediately after treatment, after 3 and after 6 months). There were no statistically significant differences (*p* > 0.05)

**Fig. 3:**
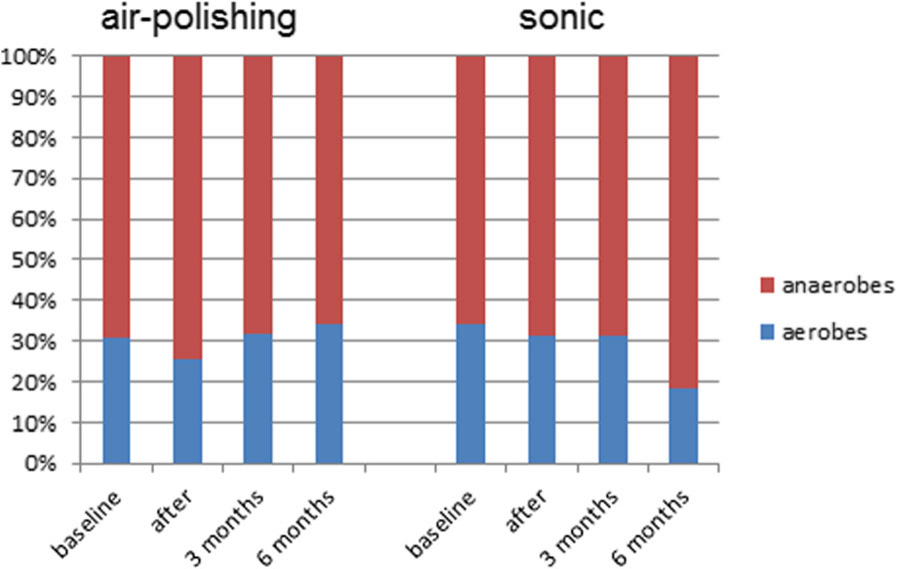
Percentage shares of aerobic and anaerobic species at different time points (baseline, immediately after treatment, after 3 and after 6 months). There were no statistically significant differences (*p* > 0.05)

The total number of aerobes was reduced after both procedures immediately after treatment compared to baseline (see Fig. 2). These increased again after three months, but remained lower than the baseline values, also after 6 months. Interestingly, the percentage of aerobes in the air-polishing group tended to be higher after six months compared to the sonic group. However, the difference between the two groups was not statistically significant. The total counts of anaerobes were lower after treatment and after 3 and 6 months for both groups (see Fig. 2). The sonic group showed an increase in the mean percentages of anaerobes immediately and 3 and 6 months after treatment with a maximum of 81.6% after 6 months (baseline 69.4%, see Fig. 3).

### Subgroups of bacterial species

**Fig. 4:**
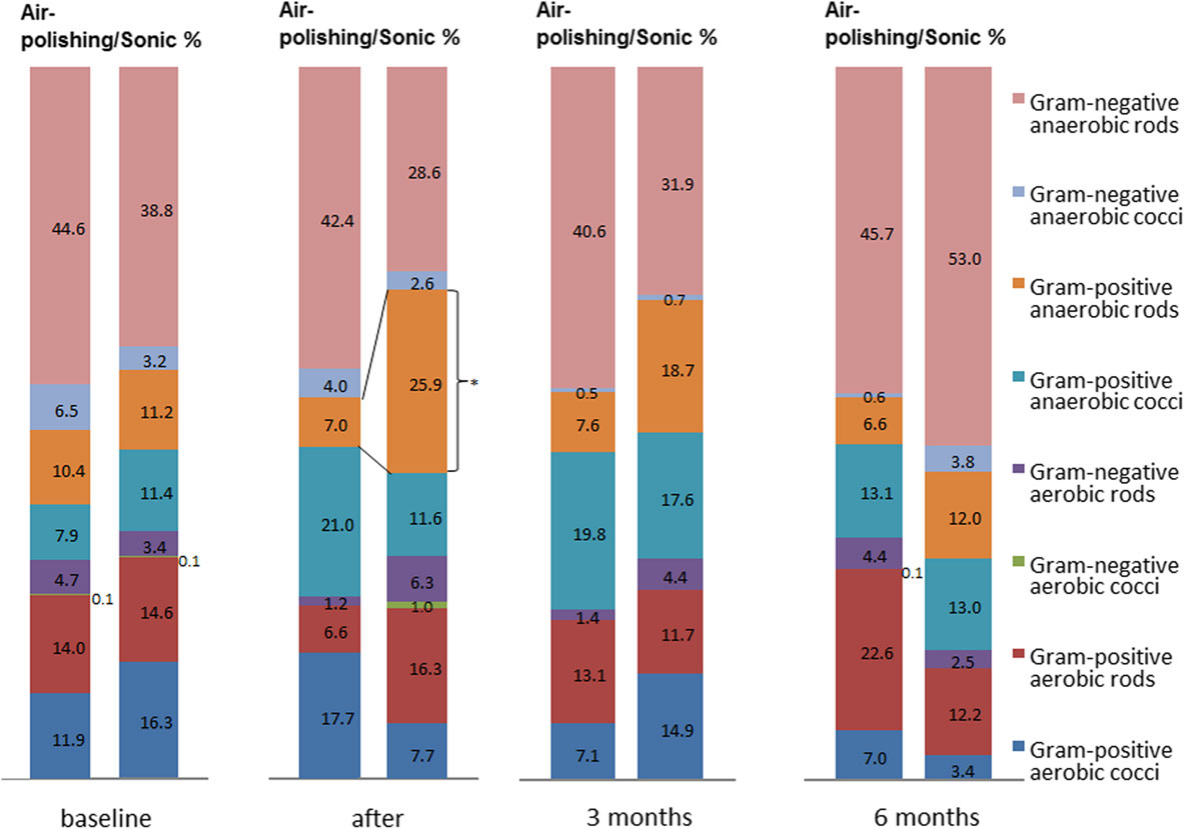
Other subgroups of bacterial species. Values are given as means in Log CFU/mL at different time points (baseline, immediately after treatment, after 3 and after 6 months). Also, percentage shares of aerobes and anaerobes are shown. Standard deviations are given where applicable, *p < 0.05

Figure 4 depicts the percentages of the main subgroups of detected aerobic and anaerobic bacterial species in the control group and in the group of patients treated with air-polishing at all four time points of microbiological sampling. The proportions of all groups including Gram-negative anaerobic rods (*Porphyromonas gingivalis*, *Prevotella intermedia*, *Prevotella nigrescens*, *Prevotella tannerae*, *Prevotella buccae*, *Fusobacterium nucleatum*, *Campylobacter rectus*, *Tannerella forsythia*, *Selenomonas* sp.), Gram-negative anaerobic cocci (*Veillonella parvula*, *Dialister pneumosintes*, *Anaeroglobus geminaus*, *Anaeroglobus geminatus*), Gram-positive anaerobic cocci (*Parvimonas micra*), Gram-negative facultative anaerobic rods (*Capnocytophaga ochracea*, *Capnocytophaga gingivalis*, *Aggregatibacter aphrophilus*, *Cardiobacterium hominis, Eikenella corrodens*), Gram-negative aerobic cocci (*Neisseria macacae/mucosa*, *Neisseria elongate*, *Neisseria flavescens*, *Neisseria bacilliformis*, *Nesseria* sp., *Lautropia mirabilis*), Gram-positive facultative aerobic rods (*Actinomyces meyeri*, *Actinomyces oris*, *Actinomyces odontolyticus*, *Actinomyces naeslundii*, *Actinomyces gerencseriae*, *Corynebacterium matruchotii*, *Rothia mucilaginosa*, *Rothia aeria*) and Gram-positive facultative anaerobic cocci (*Streptococcus oralis*, *Streptococcus mitis*, *Streptococcus sanguinis*, *Streptococcus cristatus*, *Streptococcus sinensis*, *Streptococcus salivarius*, *Streptococcus anginosus*, *Streptococcus constellatus*, *Streptococcus intermedius*, *Streptococcus mutans*, *Gemella morbillorum*, *Streptococcus* sp.) were comparable between both treatments. A significantly higher proportion of Gram-positive anaerobic rods (*Slackia exigua*, *Eubacterium yurii*, *Atopobium rimae*, *Filifactor alocis*, *Bifidobacterium dentium*, *Solobacterium moorei*, *Olsenella uli*) was detected in the patient group treated with a sonic scaler (*p* < 0.05).

### Number of different species (see Fig. 5)

The analysis of the number of different detected species showed a decrease of bacterial species. Immediately after treatment, the diversity of detected species decreased significantly more in the air-polishing group than in the sonic group (*p* = 0.03). All other differences were not statistically significant.

**Fig. 5:**
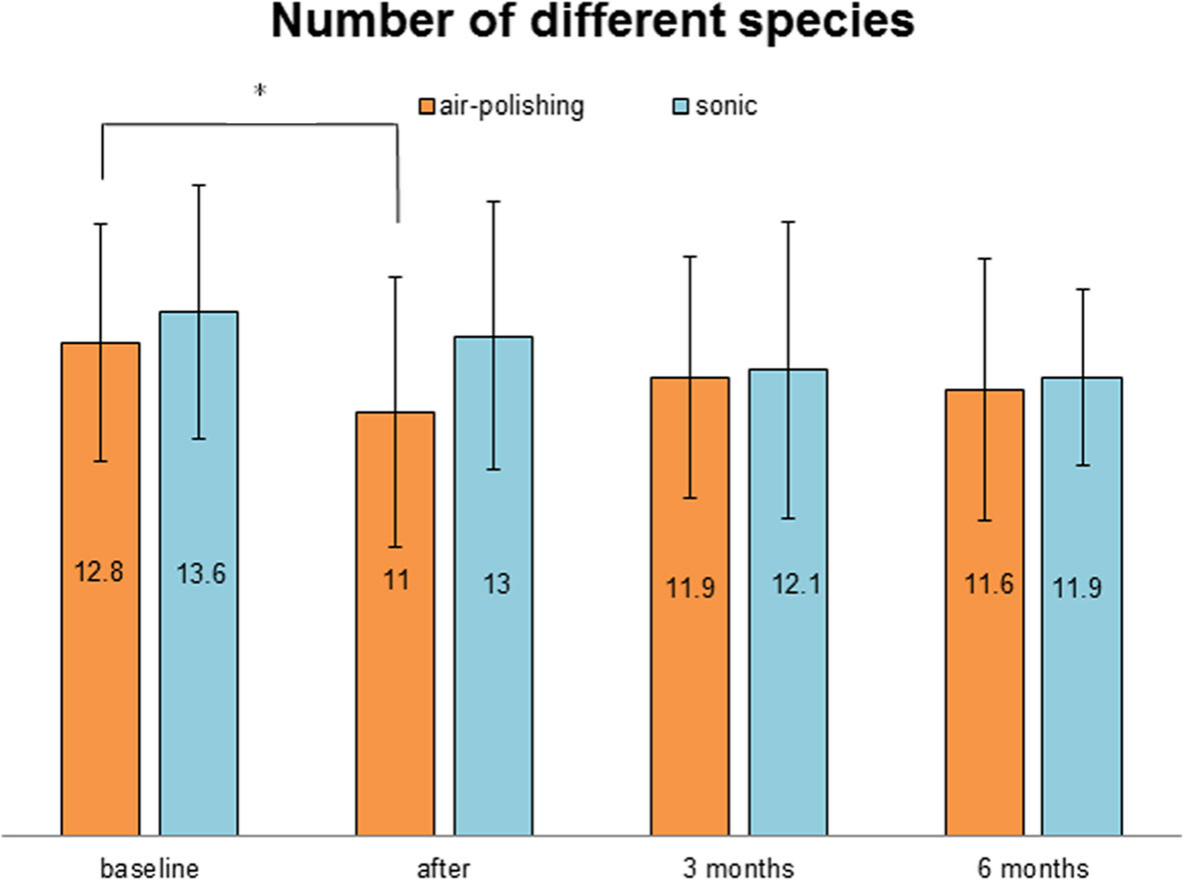
Analysis of the number of detected species. Values ​​are given in whole numbers with standard deviations (*p < 0.05)

## Discussion

This controlled randomized clinical trial investigated the effects of trehalose powder for the removal of subgingival biofilm in the context of supporting periodontal therapy over a six-month period. To the authors’ knowledge, trehalose powder was used for the first time in combination with air-polishing within a clinical trial. Furthermore, culture technique was used to draw a more differentiated picture of living and surviving bacteria in subgingival regions before and after air-polishing. So far, in similar studies this technique has been used only to determine the total bacterial load in the context of air-polishing [7, 27]. In the present study, however, the analysis of subgroups of different species as well as the analysis of aerobes and anaerobes was carried out. In contrast to this, other research groups have mostly used molecular genetic techniques and sequencing methods for the detection of previously selected bacterial species. This has the great disadvantage of not distinguishing living from dead bacteria and also limits the field of vision to a few species. The aim of the present work was to confirm the biofilm bacteria which survived both examined techniques of subgingival instrumentation. This was to evaluate the efficiency of air-polishing for the removal of the subgingival oral biofilm compared to the sonic treatment. Therefore, molecular genetic techniques such as real-time PCR, fluorescence in situ hybridization and high-throughput next generation sequencing methods have been omitted [28]. Nevertheless, such new culture independent technique would add new insight into the reestablished biofilms months after the treatment.

The results of this microbiological part of a larger study support the clinical findings [9]. Here, 10 out of 44 participants were examined for microbiological analysis. The results show that each method resulted in a significant reduction of bacteria after instrumentation and at different time points up to 6 months. No distinct differences between the two groups could be observed. However, the confidence intervals for the group differences indicate that larger studies are necessary to establish the equivalence of the two methods. The reduction of the total bacterial load after air-polishing shown in the present work agrees with the results of other studies. Moëne and co-workers [29] collected subgingival bacterial specimens two days prior to subgingival treatment with glycine powder and seven days post treatment. The authors reported a reduction in the absolute number of microorganisms, and moreover the number of six specific microorganisms (*A. actinomycetemcomitans, F. nucleatum, P. gingivalis, P. intermedia, T. denticola, T. forsythia*). However, the difference in reduction was not statistically significant compared to the use of hand instruments. In two different studies, both performed by the working group of Petersilka and co-workers, subgingival treatment was done on the buccal and lingual sites or on the interdental sites with glycine powder [7, 27]. Here, the total number of bacteria of the anaerobes before and immediately after treatment was determined. Both studies could show that a reduction in total bacterial counts after air-polishing treatment was achieved [7, 27]. Other authors have also shown that both therapies (sonic treatment and subgingival air- polishing with glycine powder) significantly reduced the periodontitis-associated species immediately and two days after treatment [6]. However, the authors did not determine the total bacterial counts, but only periodontal sites where different bacterial species had been detected were called positive sites. These increased again after 14 days and approached baseline levels before treatment. This is in accordance with the results of the present work, since after three months an increase in the total bacterial counts was observed independent of the treatment method. The observed increase in the number of total bacteria 3 months after treatment and the subsequent drop after six months may be due to the re-treatment of persistent periodontal pockets after three months. This post-treatment plays an important role in creating stable periodontal conditions and reduces tooth loss rate [30–32]. An increase in bacterial counts after three months was also described in a study by Flemmig and co-workers [11]. The authors investigated the influence of the air-polishing treatment using glycine powder on two species of the red complex (*P. gingivalis* and *T. forsythia*). The bacterial count of both bacterial species was reduced immediately after treatment, but increased again after three months and approached baseline levels.

In this study the effects on Gram-positive anaerobic rods were especially noticeable in the air-polishing group in which a higher proportion of this bacterial group was detected directly after treatment than in the patient group treated with a sonic scaler. However, the numbers of bacterial species in the air-polishing group decreased again after six months compared to the 3-month value, and were lower than the counts after the sonic treatment, but not significantly. The percentage of all aerobes and all anaerobes in the present work was almost identical in both treatment groups before treatment. A difference in this distribution between the two treatments was observed at the final 6-month follow-up, but did not reach statistical significance (*p* > 0.05, Fig. 2).

The number of different species was compared between the groups as a measure of the diversity. Changing the diversity means an alteration of the biofilm ecology and balance which may lead to disturbing of the natural biofilm functions. Such alterations have been considered also in other treatment techniques such as antimicrobial photodynamic therapy [33].

Since both treatment methods are not bactericidal and have been used to just mechanically remove the subgingival biofilm for the reduction of bacterial counts and not to kill the bacteria, no significant differences of the microbial composition of the biofilm sample over the total period of the study could be expected. Indeed, only directly after treatment was the percentage of anaerobic Gram-positive rods significantly higher in the patients group treated with sonic scaler compared to the air-polishing group. This emphasizes again the comparability of both treatment methods as to the influence on the subgingival biofilm. Hence, the variations and differences in the percentages of the main bacterial groups depicted in Fig. 4 should be considered as a natural fluctuation of the regrown subgingival biofilm in both groups. A more detailed microbial analysis using the culture technique would not impact the outcome of the microbial results presented in this study. The effects of air-polishing using trehalose powder as well as sonic treatment on the biofilm structure should be further studied using confocal laser scanning microscopy on a biofilm model generated by total salivary bacteria ex vivo on human enamel samples. This would evaluate the impact of both methods on the destruction of the oral biofilm structure and would show the residues of the biofilm on the enamel surface. One could speculate, that due to the different aspects of the technical application of both methods, the impact on the morphological appearance of the biofilm residues might be different and should be taken into consideration. While sonic scaling might only affect the upper layers of the biofilm, air-polishing may cause deeper dispersion of the biofilm leading to a stronger destruction of the biofilm. However, this has to be evaluated in morphological studies as stated above.

## Conclusions

The results of the present work suggest that air-polishing and sonic treatment have a comparable effect on the subgingival oral biofilm during supportive periodontal treatment. Moreover, the present study stressed the ability of trehalose powder to remove the subgingival biofilm mechanically. The microbiome of the regrown biofilm seems not to be affected. Yet, effects on the regrown subgingival biofilm should be studied in more detail by high throughput-sequencing in future studies.

## Supplementary information

**Additional file 1: Figure S1**. CONSORT Flow diagram

## Data Availability

The datasets used and/or analysed during the current study are available from the corresponding author on reasonable request.

## Abbreviations

°C: Degrees Celsius
A. actinomycetemcomitans: Aggregatibacter actinomycetemcomitans
BL: Baseline
BOP: Bleeding on probing
CAL: Clinical attachment level
CoBl: Columbia Blood Agar
F. nucleatum: Fusobacterium nucleatum
FISH: fluorescence in situ hybridization
HCB: Yeast cysteine ​​blood agar
MALDI-TOF-MS: Matrix-assisted laser desorption/ionization time-of- flight mass spectrometry
mL: Milliliter
P. gingivalis: Porphyromonas gingivalis
P. intermedia: Prevotella intermedia
PCR: Polymerase chain reaction
PD: Pocket depth
PY: Dilution medium
rDNA: Ribosomal deoxyribonucleic acid.
T. denticola: Tannerella denticola
T. forsythia: Tannerella forsythia
TBL: Total bacterial counts
TSBV: Trypticase Soy Bacitracin Vancomycin Selective Agar
