# Supplementary figures and images for: Effects of subgingival air-polishing with trehalose powder on oral biofilm during periodontal maintenance therapy: a randomized-controlled pilot study
Source: BMC Oral Health. 2020 Apr 22;20:123. doi: 10.1186/s12903-020-01111-9 (PMC7178568; doi:10.1186/s12903-020-01111-9)

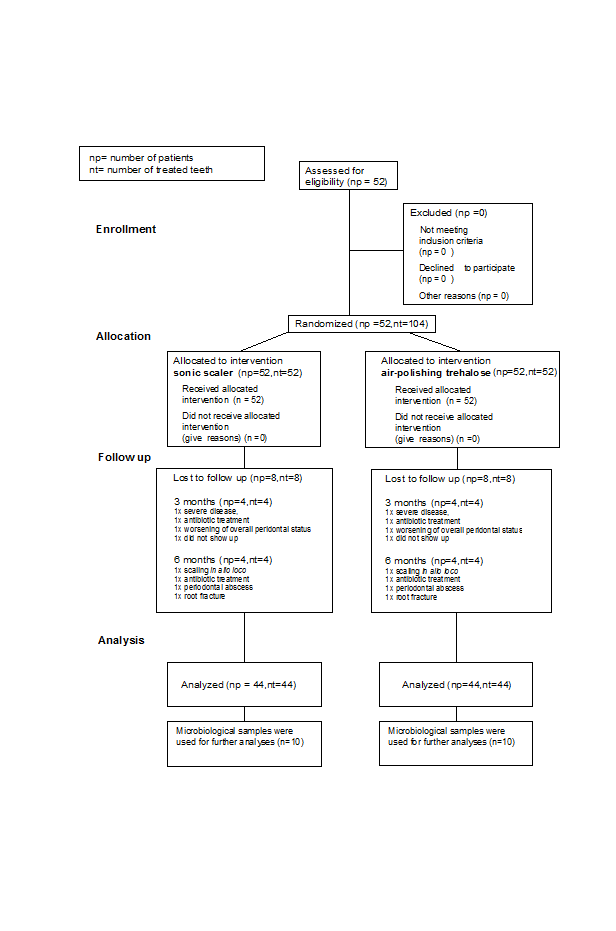

Supplement: Supplementary file 1 — Additional file 1: Figure S1. CONSORT Flow diagram [file 12903_2020_1111_MOESM1_ESM.tif]
